# Supplementary material for: Publication outcomes and research trends in bachelor’s and master’s theses in health sciences in Croatia: retrospective cohort study and survey of mentors
Source: PLoS One. 2025 Nov 3;20(11):e0335350. doi: 10.1371/journal.pone.0335350 (PMC12582455; doi:10.1371/journal.pone.0335350)
Supplement: S1 File — (DOCX) [file pone.0335350.s001.docx]

# **Supplementary file 1. Text of the survey for mentors**

[In case of having mentored multiple theses, we will create a separate file with questions for each thesis separately]

Dear XY,

You mentored a student XY on their Bachelor/Master thesis titled XY.

We are conducting the study about the publication outcomes of Bachelor and Master theses in health sciences studies in Croatia. We would appreciate if you could share with us the following information:

1. Did you publish content of the thesis in a scholarly journal?

Yes/No

If yes, please share the citation of the article with us.

If you did not publish any articles from that thesis:

2. Did you submit the article based on the thesis?

Yes/No

If yes, please share the name of the journal where you have submitted the article

If you did not submit any articles based on that thesis:

3. Do you intend to prepare an article for a scholarly journal based on that thesis?

Yes/No

If yes, what will be your target journal?

4. If you did not publish any articles from the thesis, and if you are not planning to, can you please explain your reason for that?

5. Did a student that defended this thesis ever expressed an interest to publish research reported in this thesis in a scholarly journal?

Yes/No

If Yes, could you describe what the students were suggesting?
