# Supplementary material for: Publication outcomes and research trends in bachelor’s and master’s theses in health sciences in Croatia: retrospective cohort study and survey of mentors
Source: PLoS One. 2025 Nov 3;20(11):e0335350. doi: 10.1371/journal.pone.0335350 (PMC12582455; doi:10.1371/journal.pone.0335350)
Supplement: Attachment — Submitted filename: 2025_07_10 Response to technical revision.docx [file pone.0335350.s003.docx]

Jim Lovell Santos Gonzales
PLOS ONE

Re: Manuscript revision after technical assessment

Dear Editor,

We are submitting a revised manuscript no. PONE-D-25-36339, titled Publication outcomes and research trends in Bachelor and Master theses in health sciences in Croatia: retrospective cohort study and survey of mentors.

We revised manuscript as suggested:

1. Please complete all items on the Human Participants Research Checklist that are relevant for your submission, by following this link:  <http://journals.plos.org/plosone/s/file?id=dc11/PLOSOne_Human_Subjects_Research_Checklist.docx> (Contact us at [plosone@plos.org](mailto:plosone@plos.org) if you cannot access the document.) There may be overlap between the checklist items and other queries listed below; please address any duplicated queries both in your response email and on the checklist itself. Upload the completed Human Participants Research Checklist as file type “Other” when you re-submit your manuscript. This document is for internal journal use only and will not be published if your article is accepted. The requested information will help us to assess whether your submission complies with PLOS ONE’s policies and adheres to applicable reporting standards. Note that your manuscript may be rejected if you provide incomplete or inadequate responses to the checklist questions and that changing the
‘Section/Category’ of your article does not affect this requirement.

**Author response**: We uploaded Human Participants Research Checklist, and revised the manuscript accordingly.

2. Please provide additional details regarding participant consent. In the ethics statement in the Methods and online submission information, please ensure that you have specified what type you obtained (for instance, written or verbal, and if verbal, how it was documented and witnessed). If your study included minors, state whether you obtained consent from parents or guardians. If the need for consent was waived by the ethics committee, please include this information.

**Author response**: We clarified that informed consent was obtained via email.

Sincerely,

The authors
