# Supplementary material for: Publication outcomes and research trends in bachelor’s and master’s theses in health sciences in Croatia: retrospective cohort study and survey of mentors
Source: PLoS One. 2025 Nov 3;20(11):e0335350. doi: 10.1371/journal.pone.0335350 (PMC12582455; doi:10.1371/journal.pone.0335350)
Supplement: Attachment — Submitted filename: Reply to authors BS and MS theses in HS.docx [file pone.0335350.s002.docx]

Publication outcomes and research trends in Bachelor and Master theses in health sciences in Croatia: retrospective cohort study and survey of mentors

Thank you for the opportunity to review this interesting manuscript.

Some suggestions:

1. Overall, this is an interesting and necessary study.
2. The literature review requires updating with more recent studies.
3. The study’s aim is well-articulated, targeting a significant gap - Publication outcomes and research trends in Bachelor and Master theses in health sciences in Croatia.
4. Better to separate the Strengths and limitations of this study. For instance the following is not clear if it is strength or limitation since the authors searched only 3 databases including Google and Google Scholar and did not include CINAHL and the search strategy was not clear and systematic.

49 - A large number of theses were included in the analysis (N=9861) and analysis of published

50 articles was done by surveying mentors and searching three electronic information sources.

5. In the introduction pages 68 and 69: you are limiting the interest of bachelor students to nursing research although in the title you mentioned “health sciences”

68 writing a bachelor thesis is associated with greater interest and a favourable attitude towards nursing

69 research and development.1

6. Create a concise results table:

Tighten your results with one clear table right after the narrative results so readers can scan the data fast.

7. Open the discussion with the main finding, then spell out its impact

One sentence: restate the top result.

Two to three sentences: explain how it shifts current evidence, guides future studies, EBP or changes bedside care.

Link results to implications for research and practice.

8. Rewrite the conclusion so it mirrors the results and discussion

Summarize the principal outcome and its practical meaning.

Echo any limits you noted earlier and close with a clear take-home message. Conclusion must reflect the evidence presented.

This paper has potential but needs a stronger analytical voice and updated evidence base to make a meaningful contribution.
